# Defeating Antibiotic- and Phage-Resistant *Enterococcus faecalis* Using a Phage Cocktail *in Vitro* and in a Clot Model

**DOI:** 10.3389/fmicb.2018.00326

**Published:** 2018-02-28

**Authors:** Leron Khalifa, Daniel Gelman, Mor Shlezinger, Axel Lionel Dessal, Shunit Coppenhagen-Glazer, Nurit Beyth, Ronen Hazan

**Affiliations:** ^1^Faculty of Dental Sciences, Hadassah School of Dental Medicine, Hebrew University of Jerusalem, Jerusalem, Israel; ^2^Department of Prosthodontics, Hadassah School of Dental Medicine, Hebrew University of Jerusalem, Jerusalem, Israel

**Keywords:** bacteriophages, *Enterococcus faecalis*, antibiotic-resistance, phage therapy, phage-resistance, phage cocktail

## Abstract

The deteriorating effectiveness of antibiotics is propelling researchers worldwide towards alternative techniques such as phage therapy: curing infectious diseases using viruses of bacteria called bacteriophages. In a previous paper, we isolated phage EFDG1, highly effective against both planktonic and biofilm cultures of one of the most challenging pathogenic species, the vancomycin-resistant *Enterococcus* (VRE). Thus, it is a promising phage to be used in phage therapy. Further experimentation revealed the emergence of a mutant resistant to EFDG1 phage: EFDG1^r^. This kind of spontaneous resistance to antibiotics would be disastrous occurrence, however for phage-therapy it is only a minor hindrance. We quickly and successfully isolated a new phage, EFLK1, which proved effective against both the resistant mutant EFDG1^r^ and its parental VRE, *Enterococcus faecalis* V583. Furthermore, combining both phages in a cocktail produced an additive effect against *E. faecalis* V583 strains regardless of their antibiotic or phage-resistance profile. An analysis of the differences in genome sequence, genes, mutations, and *tRNA* content of both phages is presented. This work is a proof-of-concept of one of the most significant advantages of phage therapy, namely the ability to easily overcome emerging resistant bacteria.

## Introduction

In 1967, William H. Stewart, the Surgeon General, said in the White House: “It’s time to close the books on infectious diseases, declare the war against pestilence won…,” expressing the general opinion, at the time, that antibiotics succeeded in solving all bacteria-related problems. Ironically, this very success has proved dangerous, as the frequent reliance on antibiotics has resulted in dissemination of resistant variants (Charles and Grayson, 2004; Davies and Davies, 2010).

One of the antibiotic – resistant, most frequently isolated species from hospital-associated infections, is vancomycin-resistant enterococci (VRE) (O’Driscoll and Crank, 2015; Khalifa et al., 2016). While mostly a commensal, when inhabiting the gastro-intestinal tract, Gram-positive *Enterococcus faecalis* bacterium can cause fatal infections (Rice, 2008; Orsi and Ciorba, 2013). In many cases, neither antibiotics (Erlandson et al., 2008; Chong et al., 2010) nor other antibacterial means like endodontic treatments and rinsing with calcium hydroxide, sodium hypochlorite, and chlorohexidine (Khalifa et al., 2016) have successfully prevented the morbidity or mortality resulting from *Enterococcus* infections especially in cases of biofilm (Donlan, 2001; de la Fuente-Nunez et al., 2013). The hazardous potential of these bacteria is exacerbated by the emergence of antibiotic-resistant strains, primarily VRE (Linden, 2002). So much so, that in 2013 the Center for Disease Control and Prevention (CDC) has declared VRE *enterococci* to be one amongst the top 18 drug-resistant threats to treat today^1^.

With the limitations of chemical antibiotics and the emergence of antibiotic-resistant strains, the use of bacteriophages has been regaining interest (Golkar et al., 2014). Their effective potential is magnified as they are highly strain-specific, targeting only their pathogen and tend to only minimally disrupt normal flora (Loc-Carrillo and Abedon, 2011). Moreover, bacteriophages have evolved naturally to be become the most proficient bacterial killers (Srinivasiah et al., 2008) in all forms, including hard-to-eradicate biofilms (Donlan, 2009; Khalifa et al., 2016). Use of bacteriophages has numerous other advantages over antibiotics (Loc-Carrillo and Abedon, 2011; Khalifa et al., 2016), including ease of isolation. Additionally, recent human and animal trials have shown that phages are safe for use (Burrowes et al., 2011).

We recently demonstrated the treatment potential of phages against vancomycin-resistant *E. faecalis* infections. EFDG1, an anti-*E. faecalis* phage, was isolated from sewage and its genome was characterized. This phage proved highly effective against *E. faecalis* V583 in planktonic and biofilm cultures *in vitro* and in an *ex vivo* human root canal model (Khalifa et al., 2015a).

Successful though phage therapy may be, bacterial cells resistant to phages will no doubt emerge, just as in the case of antibiotics (Duerkop et al., 2016), as indeed has occurred in our case too. However, in contrast to the grave challenges facing antibiotics, phage therapy offers several simple approaches to combat phage-resistant bacteria. One strategy would be to isolate new phages and to use them in cocktails. Based on the number of previously isolated phages and phage sequences in microbiomes, it is estimated that a bacterial strain may have as many as several 100 phages capable of combating it Khalifa et al. (2016). A second approach is to improve current phages by random or directed mutagenesis: using UV radiation (Drake, 1966) or gamma rays (Bertram, 1988), by means of chemical mutagens such as methyl methanesulfonate (MMS) (Drake, 1982) or through genetic engineering (Kiro et al., 2014; Pires et al., 2016). In any case, multiple-phage cocktails are preferable to single-phage therapy, as they are better equipped to deal with phage-resistant bacterial mutants and multispecies bacterial cultures (polyphage therapy) (Chan and Abedon, 2012; Gu et al., 2012; Ormala and Jalasvuori, 2013).

In the current article, we demonstrate the first approach: isolating a new phage against phage-resistant bacteria, constructing a phage cocktail and then utilizing it successfully against both naïve and phage-resistant mutant strains in *in vitro* models. The phage EFLK1 was effective against the EFDG1 phage-resistant *E. faecalis* strain (EFDG1^r^). Moreover, a cocktail of EFDG1 and EFLK1 phages had an additive effect on planktonic and biofilm cultures.

## Materials and Methods

### Emergence and Isolation of Phage-Resistant Bacteria

Following all experiments of EFV583 with phage, survivors were routinely tested for resistance to the phage. The survivors were plated and the resulting single colonies were collected and grown. The bacteria were then tested for susceptibility to the phage, by either spotting phages on them or by cross streak agar assay. For the spotting method, about 100–200 μl of bacterial culture was spread uniformly on a BHI agar plate and once it dried; 10 μl spots of phages were spotted, which was done in duplicates or triplicates. While the cross streak agar assays were performed by horizontally streaking the phage lysate in the middle of the plate and then vertically streaking the bacterial colonies over the phage in unidirectional streaks (Moore, 2011; Tiruvadi Krishnan et al., 2015).

Bacterial colonies that grew in presence of phage were considered to exhibit resistance to the phage. These colonies were then collected and grown at 37°C and checked numerous times for their resistant characteristics against the phage. Once their resistant property was confirmed, these bacteria were frozen with 25% glycerol at -80°C and store for future use.

### Bacterial Strains and Materials

*Enterococcus faecalis* V583 (ATCC 700802) VRE and EFDG1^r^, EFDG1 phage-resistant bacterial strain were grown in brain heart infusion (BHI) broth (Difco, Detroit, MI, United States) aerobically in an incubator shaker with shaking at 220 RPM at 37°C. BHI agar (1.5%) was used for isolation streaks and isolations of phage. For top agar, 0.6% of agar (soft agar) was used, with BHI as nutrient supplement.

### Isolation and Storage of Phage

In order to combat the phage-resistant mutant strain, a new lytic phage was isolated from the sewage effluents collected from the “Nahal Sorek” decontamination facility located in West Jerusalem, as described previously (Khalifa et al., 2015a). The effluents were centrifuged using a 5430R centrifuge, and a fixed angle rotorFA-45-24-11HS; Eppendorf at 10,000 × *g* for 10 min, to pellet out the debris. The supernatant was filtered twice, initially through 0.45-μm-pore-size filters (Merck Millipore, Ltd., Ireland) to filter out larger particles and then through 0.22-μm-pore-size filters (Merck Millipore) to achieve lysate free from any residues. Different concentrations of the filtrates (between 0.1 and 1 ml) were then added to bacterial broth culture diluted 1:1000 in BHI. Once lysis was obtained, the lysate was centrifuged and filtered using the aforementioned method.

The phage lysate was then applied on bacterial lawn by the method of double layer agar to obtain clear plaques that symbolize the presence of phages. About 100 μl of bacteria was mixed with 100 μl of phage lysate and incubated for 20 min at 37°C to achieve better adsorption of the phage to the bacteria. The entire mixture was then added to 5 ml of melted 0.6% BHI top agar, poured evenly onto a BHI agar plate and incubated overnight at 37°C. The resultant single plaques were collected and added to 1 ml BHI and incubated at 4°C for diffusion. The liquid was centrifuged at 5000 RPM for 10 min and filtered through 0.22-μm-pore-size filters to obtain a clear phage lysate that was further used to grow large quantities of the phage. The phage was grown in BHI broth with EFV583 bacteria until a titer of 10^9^ or higher was achieved. The phage titer of the phage lysate was determined by counting the Plaque forming units per ml (PFU/ml). For this; the phage lysate was serially diluted in BHI broth and 5 μl of these dilutions were spotted onto an overlayer of molten 0.6% agarose with 100 μl of bacteria. This plate was incubated overnight at 37°C and the numbers of plaques were counted to finally calculate the PFU/ml. A large volume of EFLK1 phage lysate with a high titer (10^10^) was prepared and stored at 4°C.

### Characterization of Phage Activity against *E. faecalis* Naïve and Phage-Resistant Form in Planktonic and Biofilm Cultures

To assess the phage effectiveness against planktonic bacteria, the growth kinetics were analyzed using a 96 well plate reader. The phage was added initially to both logarithmic cultures (10^4^ to 10^5^ CFU/ml) at MOI of 100–1000 (10^8^ PFU/ml) and stationary cultures (10^8^ to 10^9^ CFU/ml) of *E. faecalis* at MOI of 0.1–1 (10^8^ PFU/ml). Cell viability was also checked by determining CFU/ml for each well.

The biofilm eradication efficiency of the phage was tested by adding it in a MOI of 0.1 (10^8^ PFU/ml) to a 2-week old stationary biofilm of *E. faecalis*. To obtain a 2-week old biofilm, overnight grown bacteria were diluted with BHI to obtain 10^4^ to 10^5^ CFU/ml of bacteria, which were then plated in a 96-well plate that was incubated for 2 weeks without any change of media or addition of new bacteria. Killing efficacy was validated using viable cell counting by calculating the CFU/ml of the biofilm as described previously (Khalifa et al., 2015a). Briefly, aspirated spent media was removed from the wells followed by washing the wells twice with sterile PBS (100 ul) carefully to not disrupt the biofilm. Sterile PBS (100 ul) was added and the biofilm was scraped and collected. The cells were sonicated in a water-bath for 5–10 min followed by several dilutions (1:10) and plating on BHI plates for CFU count.

### One Step Growth

We based our protocol on the one step growth method of Ellis and Delbrück (Ellis and Delbrück, 1939). Briefly, phages were added to stationary-phase bacteria (10^9^ CFU/ml) in a final concentration of 1 × 10^7^ PFU/ml followed by incubation in 37°C for 10 min. The samples were diluted at a ratio of 1:10^4^ in fresh BHI medium, followed again by incubation at 37°C for 10 more minutes. Starting at 20 min after the introduction of the bacteriophages to the bacterial cultures, the diluted mixtures were enumerated through a double-layered agar plaque assay. To this end, 3 ml of Soft BHI Agar (0.6% Agar) were mixed with 100 μL of the diluted mixture and with 100 μL of stationary-phase bacteria, according to the tested bacterial strain. The plaque assays were conducted in the following time points after the introduction of the bacteriophages: 20, 35, 50, 60, 70, 85, 100, 120 min, in biological duplicates. Latent time and burst size were calculated from this growth curve for each of the bacteriophages described, with each of the bacterial hosts.

### Genome Sequencing

To verify that the phage is indeed new, its DNA was isolated using the Norgen Biotek Phage DNA Isolation Kit (Cat. # 46800) and the resulting genome was sequenced and analyzed (Khalifa et al., 2015a,b). Analysis and comparison to other *E. faecalis* phages was performed using the GENIOUS 10.0.6 software and its plugins^2^. Annotation of genes was carried out using PHAST^3^.

### TEM Visualization

To observe the structure of the isolated phage accurately transmission electron microscopy (TEM) by the classic method of Gill as described in OpenWetWare^4^, was conducted following the procedure mention in our previous paper (Khalifa et al., 2015a). Briefly, 1 ml of phage lysate with 10^9^ PFU/ml was centrifuged at 19,283 × *g* (centrifuge 5430R, rotor FA-45-24-11HS; Eppendorf) for 2 h at room temperature. The supernatant was discarded, and the pellet was resuspended in 200 μl of 5 mM MgSO_4_ and incubated overnight at 4°C. 30 μl of 5 mM MgSO_4_ and 10 μl of the phage sample were mixed gently on a parafilm strip and 30 μl of 2% uranyl acetate was pipetted on it. On these drops of phage samples grids were then placed carefully using forceps, with the carbon side facing down. After about a minute the grids were dried and stored in the desiccator until further use. A TEM (Joel, TEM 1400 plus) with a charge-coupled device camera (Gatan Orius 600) was used to capture images.

### Naïve and Resistant Bacterial Mixture

The naïve and resistant *E. faecalis* were mixed in 1:1 ratio (v/v) and the efficacy of phages was checked individually and in cocktail against them. The experiment mirrored the one described above (see section “Characterization of Phage Activity against *E. faecalis* Naïve and Phage-Resistant Form in Planktonic and Biofilm Cultures”) for assessing phage activity on planktonic bacteria and on biofilm.

### *In Vitro* Fibrin Clot Model

The *in vitro* fibrin clot model was prepared according to the protocol described by McGrath et al. (1994) and Entenza et al. (2009). Overnight cultures (10^9^ CFU/ml) of *E. faecalis* individually or in a mixed cultures of naïve and resistant bacteria were diluted 1:10 with citrated plasma. Clots were formed by triggering coagulation by adding 20 μl bovine thrombin (5000 U/ml) and 20 μl CaCl_2_ (50 mmol). The resultant clots were then re-suspended in 500 μl of bacteriophage lysate of 10^8^ PFU/ml and for the control, 500 μl of BHI were added instead. The tubes were kept in an incubator-shaker for 6 h at 37°C. After incubation, the clots were washed with sterile PBS and lysed with 32 μl of 0.25% of Trypsin EDTA. After a 5-min centrifugation at 14,000 rpm, the cell pellet was re-suspended in 100 μl of PBS and used for calculating CFU/ml.

## Results

### Emergence of the Phage-Resistant *E. faecalis* Mutant EFDG1^r^ and Isolation of EFLK1 Phage to Combat It

**FIGURE 1:**
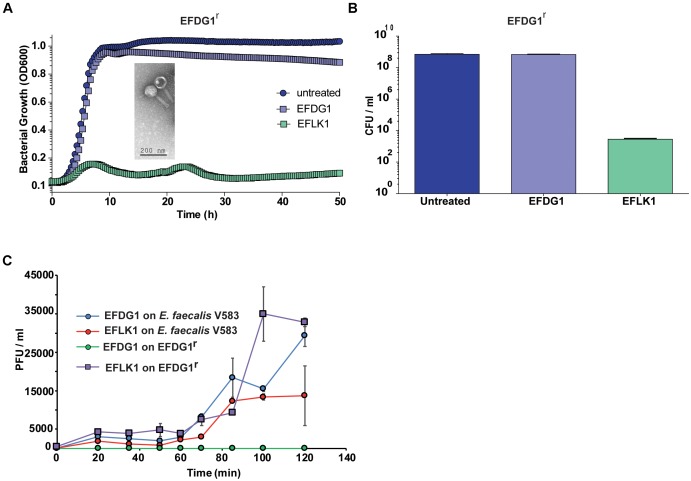
Emergence of phage-resistant bacteria, EFDG1^r^. The growth curve and CFU/ml values of EFDG1^r^ bacteria treated with EFDG1 phage resemble the untreated version of the same. Phage EFLK1 exhibited efficient lysis effect on the bacteria. **(A)** Logarithmic phase EFDG1^r^ bacteria without phage action (untreated) and with phages EFDG1 and EFLK1. The image inside the graph shows transmission electron microscopy (TEM) visualization image of the phage. **(B)** Colony counts revealing the CFU/ml of untreated and treated EFDG1^r^ bacteria after phage treatment. **(C)** One step growth curve of phages growing on and bacteria. Phages were added at 10^4^ PFU/ml. One-step growth curve of EFDG1 and EFLK1 on *Enterococcus faecalis* V583 and EFDG1^r^.

The lytic phage EFDG1 was isolated against vancomycin-resistant strains of *E. faecalis* V583 planktonic and biofilms cultures (Khalifa et al., 2015a). When strain EFDG1^r^ (resistant to EFDG1 phage) evolved during the experiments (**Figure 1A**), we screened various sewage effluent samples containing potential anti-*E. faecalis* phages. Within several weeks, a new lytic phage was isolated, EFLK1, from a sample collected from the sewage water of the “Nahal Sorek” decontamination facility. The new phage exhibited clear plaques on double-layered agar lawn of EFDG1^r^. It also prevented the growth of EFDG1^r^, as observed both in optical density growth curve (**Figure 1A**) and colony forming units (CFUs) count (**Figure 1B**). In agreement, a one-step growth experiment revealed that phage EFLK1 propagated on an EFDG1^r^ while EFDG1 phage did not (**Figure 1C**).

### Phenotypic Differences Between EFDG1 and EFLK1 Phages

**FIGURE 2:**
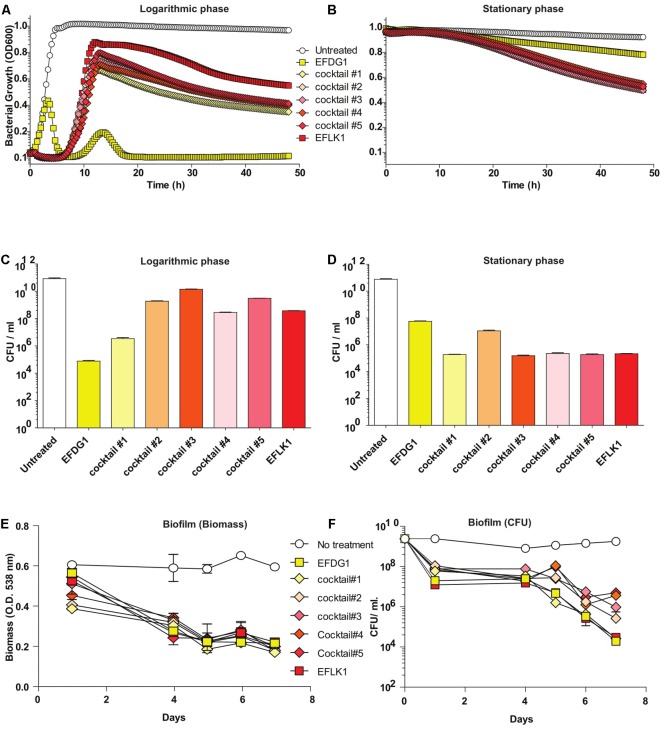
Finding the most effective phage cocktail. **(A)** Logarithmic and **(B)** stationary phase *E. faecalis* V583 treated with EFDG1, EFLK1, and cocktails of these two phages in different ratios. Cocktail#1 (1:1), Cocktail#2 (1:2), Cocktail#3 (2:1), Cocktail#4 (1:3), and Cocktail#5 (3:1). Number of colony forming units per ml (CFU/ml) for logarithmic **(C)** and stationary phase **(D)** bacteria with and without treatment of the phages and their cocktails. **(E)** Biofilm biomass quantified by crystal violet staining of 2-weeks of *E. faecalis* V583 biofilm. **(F)** CFU/ml for phage treated and untreated 2-weeks old *E. faecalis* V583 biofilm.

Despite the similarity between the two phages (32.187%), the efficiency and kinetics of EFLK1 phage infectivity against the parental strain V583 was distinct from that of EFDG1 phage. The lysis caused by EFDG1 phage during logarithmic growth was observed within 5 h (**Figure 1A**) and it reduced the viable cells number by four logs (**Figure 2A**) and (Khalifa et al., 2015a). In these conditions, EFLK1 phage was markedly slower; its lysis started after 12 h (**Figure 2A**) and it reduced cell viability by three logs (**Figure 2C**). In contrast, EFLK1 phage faired better against stationary cultures of V583 strain: EFLK1 phage lysis was observed after 10 h compared to much less significant lysis by EFDG1 phage (**Figure 2B**). These differences in lysis are reflected in the reduction of cell viability from 10^11^ to ∼10^5^ by EFLK1 phage and only to ∼10^8^ by EFDG1 phage (**Figure 2D**).

### *In Vitro* Characterization of Combinatorial Cocktails of EFDG1 and EFLK1 Phages Aimed for Therapy

Since we aim to use EFDG1 and EFLK1 phages as therapeutic agents, we tested various combinations of phage cocktails, initially on naïve *E. faecalis* V583. To this end, we combined EFDG1 and EFLK1 phages in various ratios: 1:1 (cocktail#1), 1:2 (cocktail#2), 2:1 (cocktail#3), 1:3 (cocktail#4), and 3:1 (cocktail#5) correspondingly (**Figure 2**). The best cocktail was found to be Cocktail#1 (1:1) as it killed the logarithmic culture of *E. faecalis* V583 as efficiently as EFDG1 phage (**Figures 2A–C**), and the stationary culture like EFLK1 phage (**Figures 2B–D**). Thus, cocktail#1 equaled the better phage in each case and outperformed the other cocktails against the challenging biofilm of *E. faecalis* V583 (**Figure 2E**).

**FIGURE 3:**
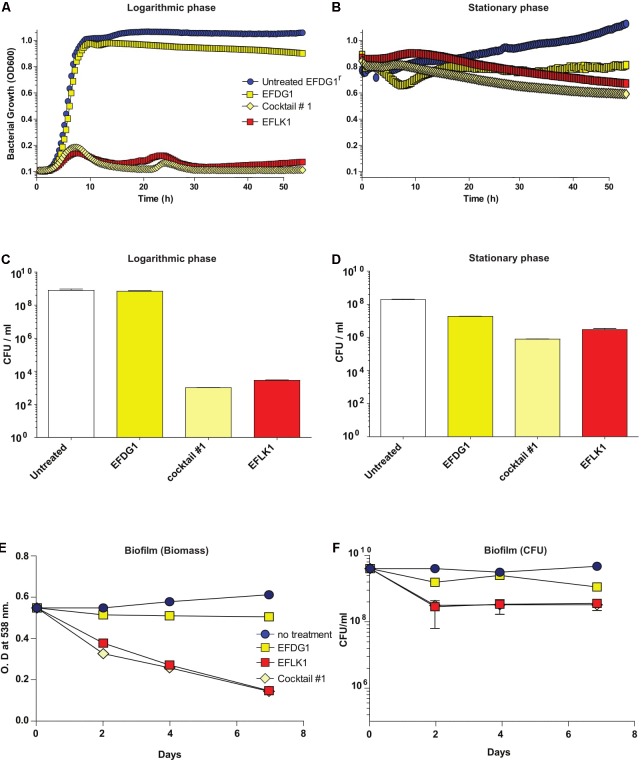
Overcoming phage-resistance. **(A)** Logarithmic growth curve and their **(C)** CFU/ml along with **(B)** stationary growth curve and their **(D)** CFU/ml values depicting the lysis effect of EFLK1 phage and phage cocktail#1 (EFDG1:EFLK1 1:1) on EFDG1^r^ bacteria, whereas EFDG1 phage has no effect at all. Similarly, reduction is seen in the biofilm biomass **(E)** and the colony count **(F)** of a 2 week old biofilm of EFDG1^r^ bacteria only when treated with EFLK1 phage or phage cocktail#1.

When cocktail#1 was used against EFDG1^r^ mutants, it was found to be as effective as EFLK1 phage against logarithmic, stationary and biofilm cultures of the resistant mutants (**Figure 3**).

**FIGURE 4:**
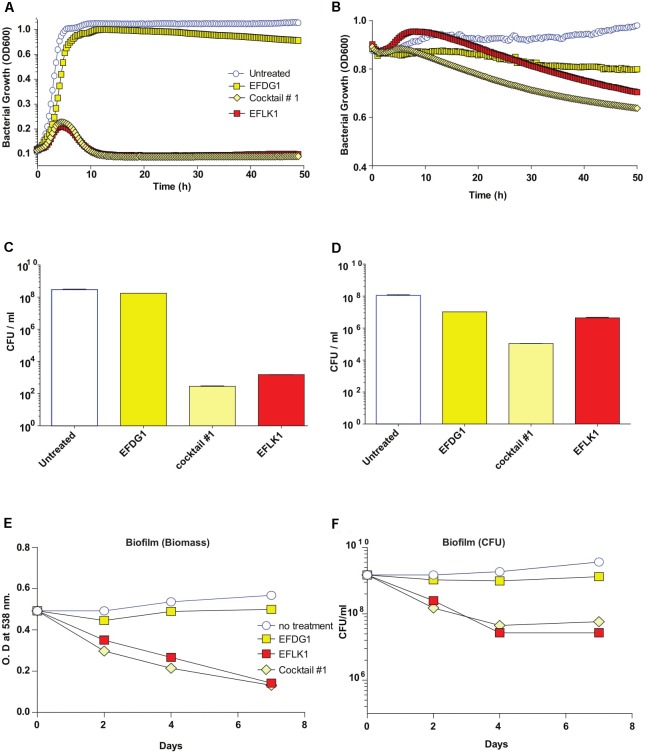
Mixed bacterial population and Phage Cocktails. Similar to EFDG1^r^ bacteria; when the naïve *E. faecalis* V583 and mutant EFDG1^r^ are mixed in equal concentrations to get a mixed population; the growth curve **(A)** for logarithmic and **(B)** for stationary phase bacterial mixtures and their CFU/ml count counterparts **(C)** and **(D)** logarithmic and stationary respectively show desirable lysis by EFLK1 phage and its 1:1 phage cocktail. While EFDG1 phage has no significant effect in either case. **(E)** Crystal violet staining reveals a reduction in biofilm biomass and **(F)** CFU/ml also shows reduction in bacterial counts when treated with EFLK1 phage and phage cocktail.

Finally, in order to simulate the evolution of resistant mutants within a culture, we mixed the WT *E. faecalis* V583 with its resistant mutant EFDG1^r^ and treated them with individual phages and cocktail #1, separately (**Figure 4**). We found that cocktail #1 and EFLK1 phage were effective against the bacterial mixture while EFDG1 phage was not.

### Demonstrating Phage Efficacy in a Fibrin Clot Model

**FIGURE 5:**
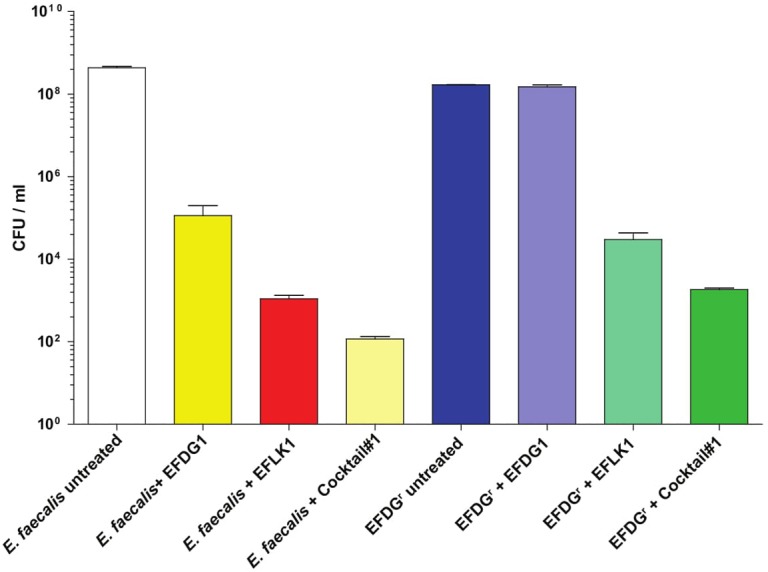
*In vitro* fibrin clot model. The inset picture shows the clot model with phage (right) and without any phage treatment (left). The clot untreated by phage disintegrates due to the presence of *EFV583* bacteria while the treated clot is intact. The number of bacterial colonies in the clot before and after phage treatment were counted and CFU/ml was calculated for each sample after clot degradation.

A recognized tool for the study of bacterial endocarditis (Hershberger et al., 2000), we used the *in vitro* fibrin-clot infection model (McGrath et al., 1994; Entenza et al., 2009) to assess the efficacy of the cocktail and the individual phages EFDG1 and EFLK1 against *E. faecalis* V583 and EFDG1^r^ (**Figure 5**). CFU/ml results following a 6-h incubation of *E. faecalis* V583 with EFLK1 phage and cocktail#1 showed a CFU reduction of three logs with EFDG1 phage and six logs with EFLK1 phage and cocktail#1. EFLK1 phage and the cocktail also reduced the CFU by five and six logs respectively, against EFDG1^r^, while, EFDG1 phage failed as expected.

### Genotypic Differences between EFDG1 and EFLK1 Phages

**FIGURE 6:**
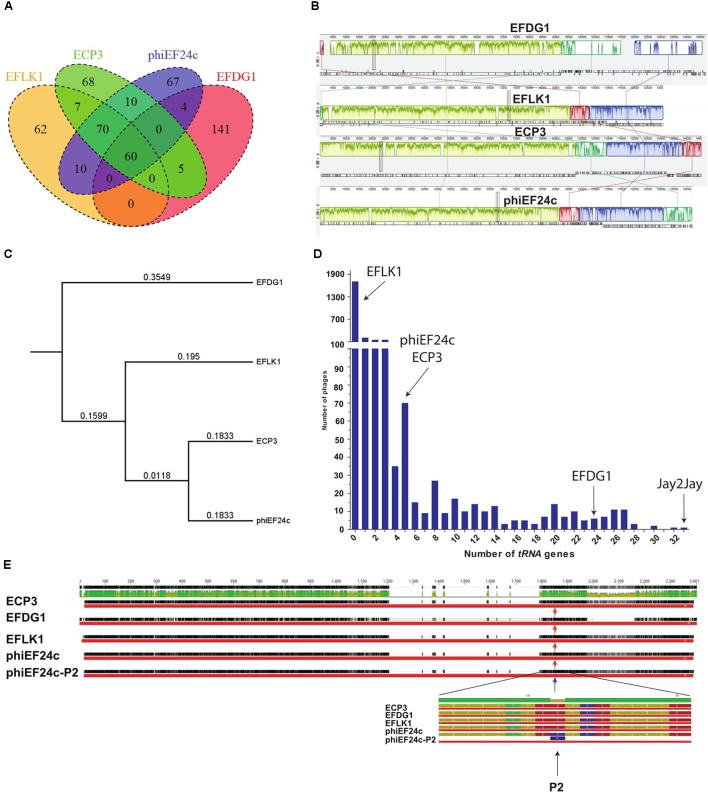
Genomic analysis. **(A)** Venn diagram represents the number of similar *tRNA* between EFLK1, ECP3, phiEF24c, and EFDG1 phages (from left to right). **(B)** Comparative analysis of the genomes of EFDG1, EFLK1, ECP3, and phiEF24c phages (top to bottom). **(C)** Phylogenetic analysis depicting the similarity between the genomes of the four known phages of *E. faecalis*. **(D)** The number of *tRNA* genes present in the genomes of almost all known phages of *E. faecalis* V583 is shown here. With EFLK1 phage **(E)** P2 site mutation is known to be linked to enhanced adsorption as shown by Uchiyama et al., 2011. No differences were found in the P2 mutation site in all the cases.

To explain the phenotypic differences between EFLK1 and EFDG1 phages, their genomes were compared (Khalifa et al., 2015a). In addition to EFLK1 phage, three additional *E. faecalis* phages of the Spounavirinae subfamily of the Myoviridae phage family of lytic phages (Lavigne et al., 2009), were described. The highest resemblance (∼45%) was found with EFDG1 phage, previously isolated by us (Khalifa et al., 2015a), and less with the other two phages phiEF24c and ECP3 (**Figures 6A–C**). All four phages share 60 core genes which are ∼28% of their genes (**Figure 6A**).

The most significant difference between EFDG1 and EFLK1 phages genome sequences found was the number of *tRNA* genes (**Figure 6D**): EFDG1 phage contains 24 of them, EFLK1 phage carries none and the other two phages (phiEF24c and ECP3) contain five each which are all clustered in one region as in EFDG1 phage (**Figure 6D**). An analysis of the distribution of *tRNA* genes among all 2,053 fully sequenced tailed phages (NCBI, Caudovirales, taxid: 28883) revealed that nearly 60% (1,227) lacked *tRNA* genes as well (**Figure 6D**). Of the remainder, the majority carried only 1–5 genes, just like ECP3 and phiEF24c (**Figure 6D**). The highest number of *tRNA* genes (33) was found in the *Streptomyces* phage Jay2Jay (accession: KM652554.1). The cassette of five *tRNA*s in phiEF24c is identical to that in ECP3. EFDG1 phage contains the same five *tRNAs* and 19 more, all in a different order. Blast of these cassettes shows that they are unique and do not correspond to the ones on the *E. faecalis* genome.

One explanation for the presence of *tRNA* genes in phages is variance in codon usage between their own genome and that of the host. Consequently, one would expect EFDG1 phage codon usage to correlate less with that of its *E. faecalis* host than the codon usage of the other phages and the large amount of *tRNAs* to compensate for that extra bias. However, COR function (R Bioconductor package) analysis disproved this theory, as no significant differences were found between the codon usage of the phages and *E. faecalis*. EFDG1 has 0.921 ECP3; 0.89522, EFLK1; 0.8954, and phiEF24c share 0.8948 correlation with the *E. faecalis* codon usage (**Supplementary Table S1**).

Next, we looked at the specific *tRNA* genes carried by these phages in accordance with their codon usage (**Supplementary Table S1**). The three anti-*E. faecalis* Spounavirinae phages with *tRNA’s* (EFDG1, ECP3, and phiEF24c) carry *tRNA* genes corresponding to codons CTA, AGA, GAC, and TGG of leucine, arginine, aspartic acid, and tryptophan respectively. Besides these, EFDG1 phage encodes for two while ECP and phiEF24c phages for single *tRNA* corresponding to the initiation and sole methionine codon AUG. Indeed, in the three phages, the usage of all these codons is higher than their usage in *E. faecalis* (**Supplementary Table S1**), perhaps justifying the corresponding *tRNA* gene’s existence. Surprisingly, the codon usage of these five codons is also higher in EFLK1 phage, which does not have any *tRNA* genes (**Supplementary Table S1**). Moreover, the codon usage should lead the four phages to carry the *tRNA* gene corresponding to codon TAC (tyrosine) while EFLK1, ECP3, and phiEF24c phages should carry *tRNA’s* for AAG (lysine), AAC (asparagine), ACA (threonine) and GTA (valine) like EFDG1 phage, however this is not the case. On the other hand, EFDG1 phage carries several *tRNA* genes, such as TTA (leucine) which, according to the codon usage, seems to be not required. Thus, the question of the effect of codon usage on the presence of these *tRNA* and its relation to the phenotypic differences remains open.

We also analyzed the P2 mutation described by Uchiyama et al. (2011) in phage phiEF24c. The P2 mutation, located in Orf31, a conserved putative tail fiber, dramatically improves the ability of phiEF24c to adsorb and lyse the cells (Uchiyama et al., 2011). Nevertheless, EFDG1, EFLK1, and ECP3 phages harbor homologs for Orf31 but none of them has the P2 mutant allele (**Figure 6E**).

## Discussion

Phage therapy holds the promise of succeeding where antibiotics have failed against resistant bacteria. In some cases, phage resistance actually has led to loss of bacterial virulence (Scott et al., 2007; Zahid et al., 2008; Capparelli et al., 2010). In addition, while production of new antibiotics is slowing down (Silver, 2011; Laxminarayan et al., 2013), the rate of new phages isolation is increasing and new phages are being discovered daily as can be seen in phage databases such as the European Nucleotide Archive (ENA^5^). It is speculated that phages are the most prevalent replicating form on Earth and their number is estimated to be ∼10^31^, which is about 10 times more than prokaryotes (Diaz-Munoz and Koskella, 2014). Thus, in case of resistance, the likelihood of isolating an appropriate phage is indeed high. Furthermore, the variety of phages supports the construction of an almost unlimited number of combinations of cocktails to combat various strains or multispecies infections.

In this work, we demonstrated the isolation of a new phage when facing phage-resistant mutants and the advantages of a two-phage cocktail against VRE *E. faecalis* and its phage-resistant mutant.

Although similar, phages EFDG1 (Khalifa et al., 2015a) and EFLK1 have interesting phenotypic differences. While EFDG1 phage is more efficient against logarithmic *E. faecalis* V583 cultures, EFLK1 phage is a better killer of stationary (**Figure 2**). Joined in cocktail, they are even better at killing the bacteria than individually. Additionally, where EFDG1 phage failed to kill EFDG1^r^, EFLK1 phage and the cocktail were effective against the WT and the phage-resistant mutant alone or in a mixed culture. This simulates the evolution of such mutants. We found the 1:1-ratio phage cocktail to be most effective, but we expect that ratios will vary and should therefore be determined on a case by case basis.

The reason for the phenotypic differences between EFDG1 and EFLK1 phages remains shrouded. One significant feature is the presence of 24 *tRNA* genes in EFDG1 phage, and their absence in EFLK1 phage. Theoretically, phages effective in the logarithmic stage (EFDG1), which benefits from elevated levels of host *tRNA*, should conceivably need to encode a small number of *tRNA* genes while phages effective in the stationary phage (EFLK1) should conceivably need a greater number. However, our findings indicated the opposite. The logarithmic efficient EFDG1 phage has more *tRNA* than the stationary efficient EFLK1 phage. Furthermore, the phages exhibited similar codon usage. In addition, the *tRNA* genes of EFDG1, ECP3, and phiEF24c phages are all encoded in a conserved region which is absent from EFLK1’s genome. This suggests that the *tRNA*’s are required for the efficient translation of the proteins encoded in that region. Though one would expect to find a higher codon usage bias with respect to *E. faecalis* in this region, this is not the case. Despite a correlation between *tRNA* abundancy and virulence against bacteria (Bailly-Bechet et al., 2007) and evidence that deletion of *tRNA* genes reduced burst sizes and protein synthesis rates in the case of phage T4 of *E. coli* (Wilson, 1973), the role of *tRNA* in phages remains enigmatic.

P2 mutation in Orf31 (**Figure 6**), found to enhance adsorption in phiEF24c (Uchiyama et al., 2011), is absent in our phages. Nevertheless, Orf31 of EFDG1 phage appears to be less conserved in general compared to the other three phages of *E. faecalis*. This could mean that EFDG1 phage binds with a different affinity, or binds to another receptor all together. Further research is needed to determine whether the differences in Orf31 are responsible for the phenotypic differences between EFDG1 and EFLK1 phages. The effectiveness and complementary nature of EFDG1 and EFLK1 phages marks these phages as suitable for *in vivo* experiments toward the development of a high-efficacy anti-*E. faecalis* phage therapy.

## Author Contributions

RH initiated the research, designed the experiments, analyzed the data, and wrote the paper. LK designed and performed the experiments and wrote the paper. MS performed some of the experiments. AD performed the bioinformatic analysis. SC-G designed the experiments and wrote the paper. DG helped to perform some of the experiments. NB initiated the research, designed the experiments, analyzed the data, and wrote the paper.

## Conflict of Interest Statement

The authors declare that the research was conducted in the absence of any commercial or financial relationships that could be construed as a potential conflict of interest.

## Footnotes

1 https://www.cdc.gov/drugresistance/biggest_threats.html

2 https://www.geneious.com

3 http://www.phantome.org

4 http://openwetware.org/wiki/Gill:Preparing_phage_specimens_for_TEM

5 http://www.ebi.ac.uk/genomes/phage.html

## Supplementary Material

TABLE S1 *tRNA* codon usage in EFDG1, EFLK1 and the other two *E. faecalis* phages as compared to that in *E. faecalis.* This is depicted by the frequency occurrence of the amino acid codon and the number of *tRNA* encoded by them in each case.
